# IL-22 dampens the T cell response in experimental malaria

**DOI:** 10.1038/srep28058

**Published:** 2016-06-17

**Authors:** Julie Sellau, Catherine Fuentes Alvarado, Stefan Hoenow, Maria Sophie Mackroth, Dörte Kleinschmidt, Samuel Huber, Thomas Jacobs

**Affiliations:** 1 Bernhard-Nocht-Institute for Tropical Medicine, Bernhard-Nocht-Straße 74, 20359 Hamburg, Germany; 2 University Medical Center Hamburg-Eppendorf, I. Department of Medicine, Martinistraße 52, 20246 Hamburg, Germany

## Abstract

A tight regulation between the pro– and anti–inflammatory immune responses during plasmodial infection is of crucial importance, since a disruption leads to severe malaria pathology. IL-22 is a member of the IL-10 cytokine family, which is known to be highly important in immune regulation. We could detect high plasma levels of IL-22 in *Plasmodium falciparum* malaria as well as in *Plasmodium berghei* ANKA (PbA)-infected C57BL/6J mice. The deficiency of IL-22 in mice during PbA infection led to an earlier occurrence of cerebral malaria but is associated with a lower parasitemia compared to wt mice. Furthermore, at an early time point of infection T cells from PbA-infected *Il22*^−/−^ mice showed an enhanced IFNγ but a diminished IL-17 production. Moreover, dendritic cells from *Il22*^−/−^ mice expressed a higher amount of the costimulatory ligand CD86 upon infection. This finding can be corroborated *in vitro* since bone marrow-derived dendritic cells from *Il22*^−/−^ mice are better inducers of an antigen-specific IFNγ response by CD8^+^ T cells. Even though there is no IL-22 receptor complex known on hematopoietic cells, our data suggest a link between IL-22 and the adaptive immune system which is currently not identified.

An important element of immune-regulatory mechanisms is the IL-10 cytokine family, which includes IL-22, a member of the IL-20 subfamily^1^. The characteristic feature of this subfamily is their ability to link the immune system with epithelial tissues. The IL-20 cytokines are mainly produced by cells of the immune system. However, the expression of the receptors is only shown on non-hematopoietic cells so far^2^, nevertheless recent studies were able to show an IL-22Rα1 expression on hematopoietic cells in autoimmunity diseases^3^,^4^. The IL-22 receptor is heterodimeric and is composed of the IL-22Rα1 chain and the IL-10Rβ chain. The binding of IL-22 to its receptor induces phosphorylation of the kinases Jak1 and Tyk2 followed by the phosphorylation of the transcription factors STAT1, STAT3 and STAT5 5,6 and leads to the maintenance of the homeostasis of epithelial cells at barrier surfaces^7^,^8^. The interaction of IL-22 with its receptor can be inhibited by a soluble IL-22 binding protein (IL-22BP or IL-22Rα2). IL-22BP is mainly expressed in mucosal tissues and in the spleen^9^. The IL-22BP-producing cell type is not exactly identified even though first findings show that CD103^+^ CD11b^+^ dendritic cells in the intestinal lamina propria produce IL-22BP under homeostatic conditions in response to retinoic acid^9^,^10^. Hence, the function of IL-22 on IL-22Rα1 expressing cells is controlled by the soluble IL-22BP, which has a higher binding affinity to IL-22 than IL-22Rα1^11^. IL-22 is produced by different cells of the immune system including T, B, NK, γδ^+^ T cells and also ILC 3 cells^12^,^13^. IL-6, IL-21 and IL-23 are STAT3 activating cytokines which are able to induce IL-22 production by T cells and ILC 3 cells^14^,^15^,^16^. Other important pathways known to induce IL-22 in T cells are the Notch and aryl hydrocarbon receptor signaling^17^,^18^. The function of IL-22 in immunity is controversially discussed. In psoriasis the expression of IL-22 is increased; it promotes inflammation, epidermal hyperplasia and additionally induces chemokines and pro-inflammatory cytokines^19^. In inflammatory bowel disease IL-22 is highly expressed as well but has both protective and pathogenic effects, depending on the type of colitis^20^,^21^,^22^,^23^. In the liver, where the IL-22Rα1 chain is highly expressed on hepatocytes, IL-22 has protective effects^24^. Furthermore, IL-22 is known to play a role concerning the composition of the microbiota^25^, especially during infection^26^. Moreover, IL-22 is involved in antimicrobial host defence by the induction of antimicrobial peptides^27^,^28^,^29^.

Since IL-22 plays an elusive role in different immune disease or infection models it is of special interest to elucidate its role in malaria. An important finding concerning IL-22 in malaria was done by Koch *et al.*^30^. *S*ince the *IL22* gene is located in proximity to the *IFNG* gene which is highly important for malaria, it was taken into consideration for further investigations regarding the severity of malaria symptoms in a large case-control study. Two haplotypes of *IL22* were found to induce either resistance or susceptibility to *Plasmodium falciparum* (*P. falciparum*) infection. This study brought attention to IL-22 for further investigations concerning its function in plasmodial infection. Further observations described that IL-22 plays a protective role during *Plasmodium chabaudi* (*P. chabaudi*) infection in mice^31^.

Besides *P. chabaudi* infection of mice there are three additional murine plasmodial species known to infect rodents: *Plasmodium berghei*, *Plasmodium yoelii* and *Plasmodium vinckei*. A widely established plasmodial model to investigate cerebral malaria symptoms is the infection of C57BL/6 mice with *Plasmodium berghei* ANKA (PbA)^32^. The plasmodial life cycle is divided into two stages: the asymptomatic liver stage and the blood-stage during which severe complications such as cerebral malaria can occur. Compared to the other strains, PbA is the most similar to *P. falciparum* concerning nucleotide identity (70.3%) and protein identity (62.9%)^33^ and causes similar symptoms in mice compared to the *P. falciparum* infection in human during the blood-stage^32^. *P. falciparum* infections still cause the majority of complicated malaria cases^34^. Cerebral malaria is the most severe complication of a plasmodial infection and can lead to death^35^. Especially in sub-Saharan Africa *P. falciparum* infections are one of the leading causes of morbidity and mortality of the disease. With the murine model of PbA infection in C57BL/6 mice the importance of the immune system for disease progression was highlighted. Cerebral malaria in this model was shown to be highly promoted by CD8^+^ T cells that adhere to activated brain endothelium as a consequence of T_H_1-dependent IFNγ production^32^. Especially IFNγ was shown to play an important role concerning the development of cerebral malaria during PbA infection^36^,^37^. To keep a well-balanced immune response to plasmodial infection the induction of regulatory mechanisms during malaria is indispensable, since the disruption of this balance leads to pathology. Important for these regulatory mechanisms are the cytokines TGFβ and IL-10^38^,^39^,^40^,^41^.

In this study, blood-stage infection of C57BL/6 mice with PbA was used to analyse the impact of IL-22 during malaria. During the blood-stage of PbA infection mice start to develop cerebral malaria symptoms at d6–8 post infection (p.i.). Here we could observe that *Il22*^−/−^ mice showed a significantly earlier occurrence of cerebral malaria symptoms accompanied by a lower parasitemia compared to wt mice during PbA infection. In addition to this phenotype an early increase of IFNγ and an overall lower IL-17 production was observed in the absence of IL-22 during PbA infection. Taken together IL-22-deficient mice showed a stronger pro-inflammatory immune response during PbA infection resulting in an earlier occurrence of cerebral malaria symptoms but also resulting in lower parasitemia.

## Results

### Elevated levels of IL-22 in malaria

**Figure 1:**
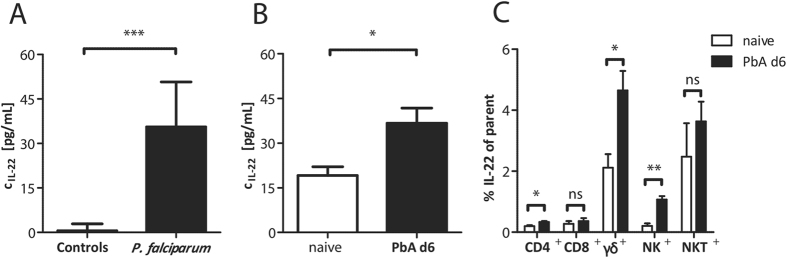
Increased IL-22 levels in plasmodial infections. Plasma samples of patients with *P. falciparum* infection were compared to healthy controls (n_control_ = 9; n_*P. falciparum*_ = 30) (**A**). Plasma samples of PbA iRBC-infected wt mice were compared to PBS treated control mice at d6 p.i. (n_control_ = 43; n_PbA_ = 36) (**B**). The concentration of IL-22 in the plasma was analysed by ELISA. IL-22 producing cells were determined by flow cytometry. For this purpose wt mice were infected with 1 × 10^5^ PbA iRBC i.p. or treated with PBS as control. At d6 p.i. mice were sacrificed and 2 × 10^6^ splenocytes were stimulated for 6 h with PMA/Ionomycin, including the last 5 h with Monensin. The percentages of IL-22 secreting cells were determined by flow cytometry and are depicted in relation the their parent cell populations (n_naive_ = 3; n_PbA d6_ = 5). Depicted are the means ± SEM.

IL-22 is significantly increased in plasma samples of human individuals suffering from acute *P. falciparum* malaria compared to healthy controls (Fig. 1A). Also in the plasma of PbA-infected C57BL/6 mice, elevated levels of IL-22 at d6 p.i. were observed (Fig. 1B). Since the IL-22 level in plasma is increased in malaria, it was of interest to specify the cell type which is responsible for IL-22 production. To this end, C57BL/6 mice were infected with PbA iRBC and sacrificed at d6 p.i. Spleen cells were stimulated *ex vivo*, stained intracellularly for IL-22 and were subsequently analysed by flow cytometry. Even though the secretion of IL-22 was significantly increased in CD4^+^ T cells as well as in NK cells, the highest proportion of IL-22 secreting cells was observed in the γδ^+^ T cell population (Fig. 1C).

### Lack of IL-22 results in a more severe malaria outcome

**Figure 2:**
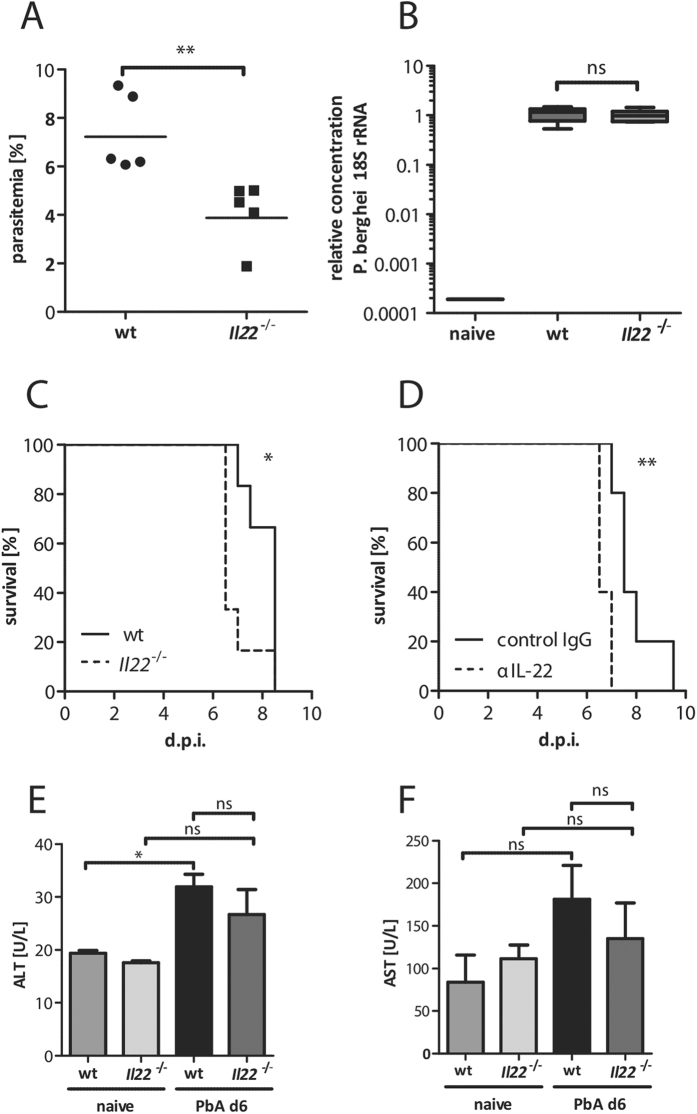
Impact of IL-22 in the course of PbA infection. Wt and *Il22*^−/−^ mice were infected with 1 × 10^5 ^PbA iRBC i.p.. The parasitemia was analysed at d6 p.i. (n_wt_ = 6; 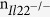 = 6) (**A**). Wt and *Il22*^−/−^ mice were infected with 1000 sporozoites i.v. The parasite burden in the liver was determined 24 h after sporozoite infection by qPCR (n_wt_ = 5; 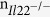 = 6) (**B**). Survival of PbA iRBC-infected wt and *Il22*^−/−^ mice were monitored during the course of infection (n_wt_ = 6; 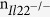 = 6) (**C**). Wt mice were infected with 1 × 10^5 ^PbA iRBC i.p. and simultaneously treated with 20 μg αIL-22 or control IgG, subsequently the course of infection was monitored (n_wt_ = 5; 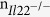 = 5) (**D**). ALT (**E**) and AST (**F**) levels in the plasma were analysed in the control groups and in PbA iRBC-infected wt and *Il22*^−/−^ mice at d6 p.i. (n_wt naive_ = 2; 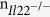
_naive_ = 2; n_wt PbA d6_ = 5; 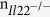
_PbA d6_ = 5). Depicted are the means ± SEM.

The infection with PbA sporozoites in the absence of IL-22 resulted in a lower parasitemia (Fig. 2A). To elucidate if this decreased parasitemia was due to a reduced parasite burden in the liver in the absence of IL-22, wt and *Il22*^−/−^ mice were infected with 1000 sporozoites i.v. and the liver load of PbA parasites was determined by qPCR 24 h p.i. (Fig. 2B). However, no differences were observed regarding the parasite burden in the liver. Hence, the effect of IL-22 on the parasitemia seems to be restricted to the blood-stage. To further analyse how the absence of IL-22 is influencing the outcome of disease, wt and *Il22*^−/−^ mice were infected with PbA iRBC and the course of the infection was monitored (Fig. 2C). Interestingly, the lack of IL-22 led to an earlier occurrence of cerebral malaria (CM). A similar result was observed by transiently neutralizing IL-22 with a specific antibody in wt mice compared to IgG treated control mice (Fig. 2D). In order to control if the lack of IL-22 during PbA infection is associated with increased inflammation in the liver ALT (Fig. 2E) and AST (Fig. 2F) levels were analysed in PbA iRBC-infected wt and *Il22*^−/−^ mice at d6 p.i. Even though an induction of ALT was observed in PbA iRBC-infected wt mice compared to the naive group, there were no differences visible during PbA iRBC infection between wt and *Il22*^−/−^ mice.

### Lack of IL-22 induces a decreased parasitemia during PyNL infection in mice

**Figure 3:**
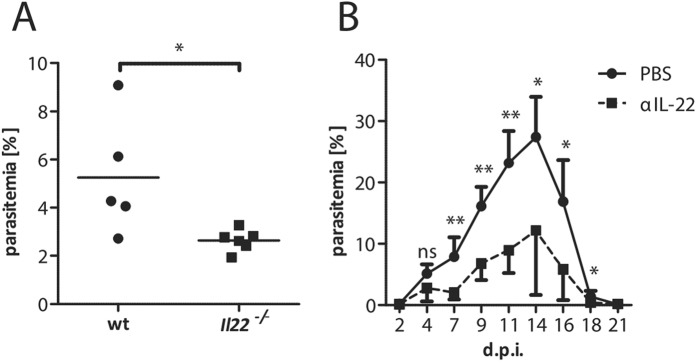
Impact of IL-22 on the parasitemia during *P. yoelii* NL infection. Wt and *Il22*^−/−^ mice were infected with 2 × 10^4^ *P. yoelii* NL iRBC i.p. The parasitemia was analysed at d7 p.i. (n_wt_ = 5; 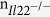 = 6) (**A**). Wt mice were infected with 2 × 10^4^ *P. yoelii* NL iRBC i.p. and simultaneously treated with 20 μg of αIL-22 or PBS as control (n_control_ = 5; n_αIL-22_ = 5). The parasitemia curve was analysed over a time period of three weeks (**B**). Depicted are the means ± SD.

In order to follow the outcome of IL-22-deficiency on the parasitemia for a longer time period, we used *Plasmodium yoelii* non-lethal (PyNL) infection of C57BL/6 mice. This *Plasmodium* species led to a transient parasitemia, which is cleared within three weeks without inducing cerebral pathology. As in the PbA model, PyNL-infected *Il22*^−/−^ mice suffer from a decreased parasitemia at d7 p.i. compared to PyNL-infected wt mice (Fig. 3A). Furthermore, the administration of a neutralizing IL-22 antibody at the time point of infection revealed a significantly decreased parasitemia compared to the control group over the course of infection (Fig. 3B).

### Increased T_H_1 response in *Il22* ^−/−^ mice during PbA infection

**Figure 4:**
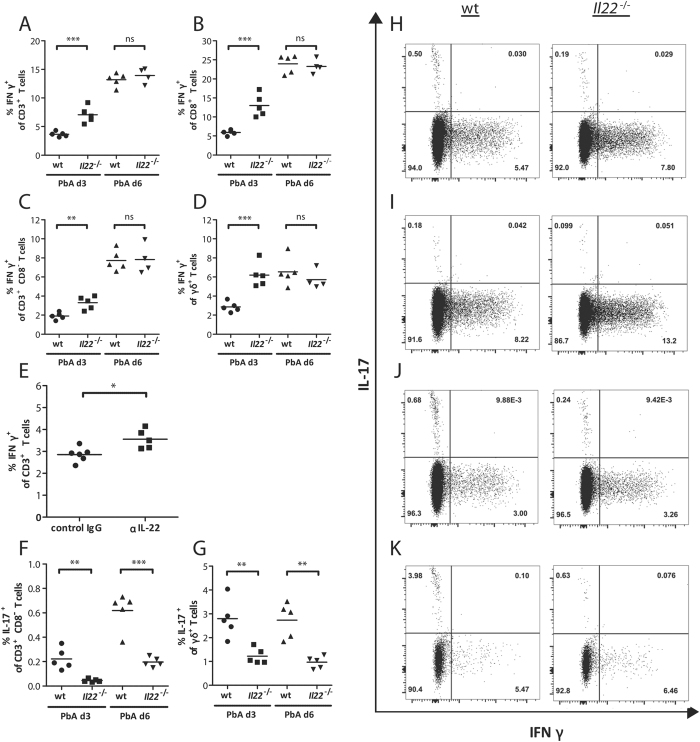
IFNγ and IL-17 secretion in PbA infection in the absence of IL-22. Splenic cells were isolated from PbA-infected wt and *Il22*^−/−^ mice at d3 (n_wt_ = 5; 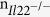 = 5) and d6 (n_wt_ = 5; 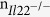 = 4/5) p.i.. These splenocytes were stimulated for 4 h with PMA/Ionomycin including the last 3 h with Monensin. The gated single cell populations of lymphocytes were additionally gated for CD3^+^ (**A,H**), CD8^+^ (**B,I**), CD3^+^ CD8^−^ (representing CD4^+^ T cells, since the CD4 receptor is internalized upon stimulation) (**C,F,J**), and γδ^+^ T cells (**D,G,K**). These populations were subsequently analysed for IFNγ and IL-17 secretion by flow cytometry. The IFNγ secretion by CD3^+^ T cells was additionally analysed from anti-IL-22 treated and PbA iRBC-infected mice and compared to the IgG treated control mice. Depicted are the means.

**Figure 5:**
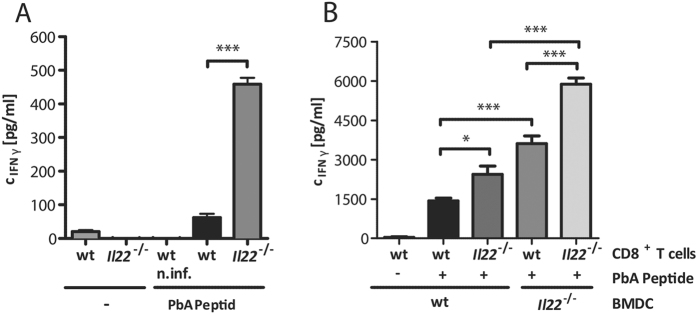
IFNγ secretion of PbA peptide stimulated splenocytes and CD8^+^ T cells of PbA-infected mice. Wt and *Il22*^−/−^ mice were infected with 1 × 10^5^ PbA iRBC. At d6 p.i. mice were sacrificed and the splenocytes were stimulated with 1 μg/mL PbA peptide for 24 h. The IFNγ concentration in the supernatant was analysed with ELISA (n_wt; w/o_ = 4; 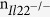
_; w/o_ = 4; n_wt n.inf; PbA Peptide_ = 4; n_wt; PbA Peptide_ = 4; n
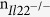
_; PbA Peptide_ = 4) (**A**). Wt and *Il22*^−/−^ mice were infected with 1 × 10^5 ^PbA iRBC. At d6 p.i. mice were sacrificed and splenic CD8^+^ T cells were purified. CD8^+^ T cells of *Il22*^−/−^ or wt mice were subsequently incubated on previously PbA peptide pulsed *Il22*^−/−^ or wt BMDCs. After an incubation time of 24 h the IFNγ concentration in the supernatant was analysed with an ELISA (n_wt CD8+; w/o PbA Peptide; wt BMDC_ = 8; n_wt CD8+; PbA Peptide; wt BMDC_ = 6; 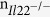
_CD8+; PbA Peptide; wt-BMDC_ = 6; n_wt CD8+; PbA Peptide; *Il22*_^−/−^_BMDC_ = 8; 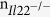
_CD8+; PbA Peptide; *Il22*_^−/−^_BMDC_ = 8) (**B**). Depicted are the means ± SEM.

Due to the increased incidence of CM in IL-22-deficient mice and since it is known that the disruption of the tight regulation between pro-inflammatory and anti-inflammatory response during malaria leads to a severe pathology, the secretion of the pro-inflammatory cytokines IFNγ and IL-17 by T cells in *Il22*^−/−^ mice was analysed. Therefore, mice were infected with PbA iRBC and splenic cells were stimulated *ex vivo* with PMA/Ionomycin. An increased level of IFNγ producing cells was observed in *Il22*^−/−^ mice compared to wt mice at d3 p.i. This effect was visible in all analysed cell types including CD3^+^ (Fig. 4A,H), CD8^+^ (Fig. 4B,I), CD3^+^ CD8^−^ (Fig. 4C,J) and γδ^+^ (Fig. 4D,K) T cells. In contrast, the IL-17 secretion was decreased in CD3^+^ CD8^−^ (Fig. 4F,J) and γδ^+^ (Fig. 4G,K) T cells at d3 p.i., which might be due to the antagonistic effect of IFNγ on IL-17 prodcution^42^. The increased IFNγ secretion at d3 post PbA infection by CD3^+^ T cells was confirmed in mice in which IL-22 was transiently neutralized compared to the control group (Fig. 4E). Even though the secretion of IFNγ was not different on d6 p.i., the IL-17 secretion of *Il22*^−/−^ mice was still significantly decreased in CD3^+^ CD8^−^ (Fig. 4F) and γδ^+^ (Fig. 4F) T cells. Nevertheless, the IFNγ concentration in the supernatant of antigen specific stimulated splenocytes from PbA-infected *Il22*^−/−^ mice at d6 p.i. was still significantly increased compared to splenocytes from PbA-infected wt mice (Fig. 5A). To gain a closer look at the mechanism behind this, bone marrow derived dendritic cells (BMDCs) from wt and *Il22*^−/−^ mice were generated and used as antigen presenting cells (APCs). CD8^+^ T cells of PbA iRBC-infected wt and *Il22*^−/−^ mice were incubated with PbA antigen-pulsed BMDCs for 24 h and subsequently the IFNγ secretion in the supernatant was analysed using ELISA. This approach showed that CD8^+^ T cells from *Il22*^−/−^ mice secrete more IFNγ after *ex vivo* antigen specific stimulation and furthermore also BMDCs from *Il22*^−/−^ mice showed an improved stimulatory effect on wt and *Il22*^−/−^ CD8^+^ T cells compared to wt BMDCs regarding the ability to induce IFNγ secretion by CD8^+^ T cells (Fig. 5B).

### Modified antigen presentation in the absence of IL-22

**Figure 6:**
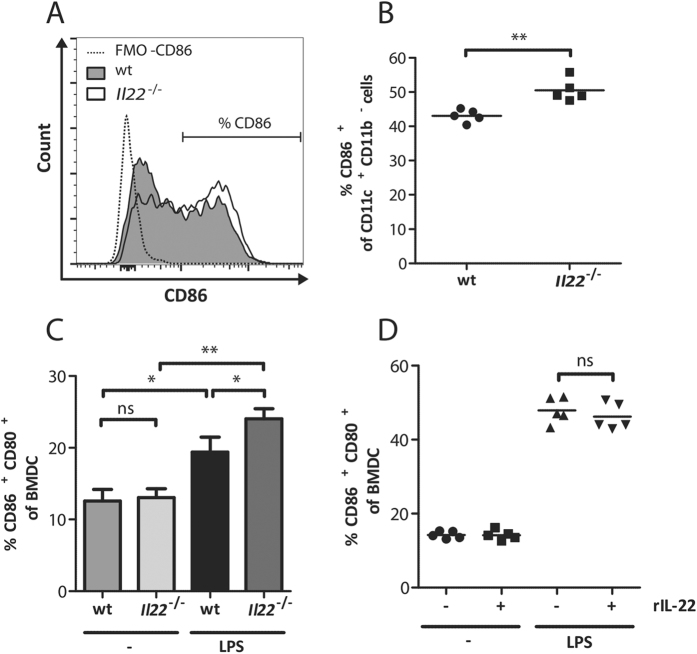
Increased induction of costimulatory receptors on dendritic cells in the absence of IL-22. PbA-infected wt and *Il22*^−/−^ mice were sacrificed at d3 p.i. and splenic cells were isolated. The exemplary histogram shows the CD86 expression of CD11c^+^ CD11b^−^ splenocytes from wt and *Il22*^−/−^ mice in comparison to the fluorescence minus one control for CD86 (FMO-CD86) (**A**). CD11c^+^ CD11b^−^ splenocytes from wt and *Il22*^−/−^ mice were analysed regarding their CD86 expression (n_wt_ = 5; 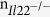 = 5) (**B**). Indicated are the means. BMDCs were stimulated with 0.1 μg/mL LPS for 24 h. The induction of CD86 and CD80 expression was determined by comparing the untreated samples with the LPS stimulated samples (**C**). The mean of four independent experiments is shown. Depicted are the means ± SEM. BMDCs of wt mice were stimulated with 0.1 ng/mL LPS in the presence of 0.05 μg/mL rIL-22 and the expression of CD86 and CD80 on the pre-gated lymphocyte population was determined (n_−;−_ = 5; n _−;rIL-22_ = 5; n_LPS;-_ = 5; n _LPS;rIL-22_ = 5) (**D**). Indicated are the means.

**Figure 7:**
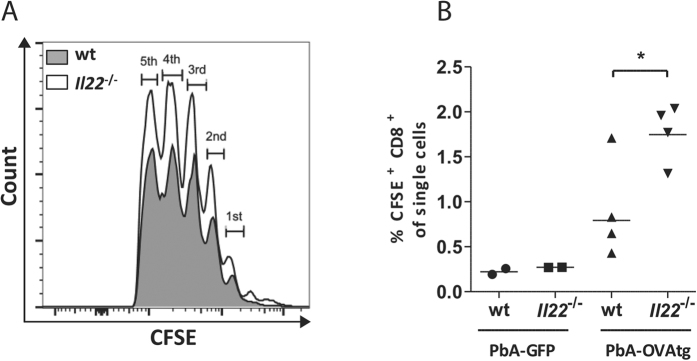
Proliferation of CFSE labelled OT1 splenocytes in wt and Il22^−/−^ mice. Splenocytes were isolated from OT1 mice, labelled with CFSE and subsequently transferred in wt and *Il22*^−/−^ recipient mice. After 3 hours these mice were either infected with PbA-GFP as a control or PbA-OVAtg. Mice were sacrificed at d5 p.i. and subsequently the proliferation of CFSE^+^ CD8^+^ lymphocytes (CFSE dilution) (**A**) and the relative amount of CFSE^+^ cells were analysed out of CD8^+^ lymphocytes (n_wt PbA-GFP_ = 2; 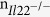
_PbA-GFP_ = 2; n_wt PbA-OVAtg_  = 4; 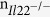
_PbA-OVAtg_ = 4) (**B**). Depicted are the means.

To elucidate why BMDCs from *Il22*^−/−^ mice have an improved ability to stimulate IFNγ secretion by CD8^+^ T cells, the expression of costimulatory ligands on antigen presenting cells from the spleen were analysed during PbA infection. For this purpose wt and *Il22*^−/−^ mice were infected with PbA iRBC and CD11c^+^CD11b^−^ dendritic cells were analysed for their CD86 expression at d3 p.i. (Fig. 6A). Dendritic cells from IL-22-deficient mice express significantly more CD86 compared to dendritic cells from wt mice at this early time point of infection (Fig. 6B). This result was confirmed by the restimulation of BMDCs with LPS over 24 h. In this case the expression of CD86 and CD80 was significantly higher after stimulation on BMDCs from *Il22*^−/−^ mice compared to BMDCs from wt mice (Fig. 6F). However, recombinant IL-22 was unable to inhibit LPS-induced expression of CD86 and CD80 on BMDCs (Fig. 6D). Since T cells are stronger activated in *Il22*^−/−^ mice during PbA infection it was of interest to analyse if the proliferation of T cells in *Il22*^−/−^ mice is altered *in vivo*. Therefore, splenic OT1 T cells were labelled with CFSE and transferred into *Il22*^−/−^ and wt recipient mice. Subsequently the recipients were infected with a transgenic OVA expressing PbA strain (PbA-OVAtg) or with a transgenic GFP expressing PbA strain (PbA-GFP) as control. Even though the proliferation of CFSE^+^ CD8^+^ T cells was highly synchronous in *Il22*^−/−^ and wt mice (Fig. 7A), the lack of IL-22 resulted in an increased fraction of CD8^+^ T cells that start to proliferate. Hence this led to a significant increase in the recovery of proliferating OT1 T cells in *Il22*^−/−^ recipient mice after infection with PbA-OVA on d5 p.i. (Fig. 7B).

## Discussion

In a previous study Koch *et al.* discovered two IL-22 haplotypes which influence the severity of malaria in West African patients^30^. The aim of the present study was to further elucidate the function of IL-22 using experimental malaria models. We find elevated level of IL-22 in the blood plasma of PbA-infected mice. Similar IL-22 levels are present in the blood plasma of *P. falciparum*-infected human individuals, further supporting the relevance of this cytokine in malaria.

The infection of C57BL/6 mice with PbA is one of the closest models to mimic the pathology of *P. falciparum* induced cerebral malaria in humans^32^,^43^. Hence this murine model was applied to elucidate the effect of IL-22 on the outcome of malaria. Here we could show that the absence of IL-22 in murine malaria exacerbates cerebral malaria symptoms. That IL-22 plays a protective role was already found during *P. chabaudi* infection^31^. Also in other diseases like multiple sclerosis^44^ or hepatitis^24^,^45^ IL-22 were shown to exert protective functions. During a *P. chabaudi* infection CD4^+^ and CD8^+^ T cells were shown to produce IL-22^31^, whereas in a PbA infection γδ^+^ T cells beside others showed the highest ability to produce IL-22. γδ^+^ T cells are known to play a crucial role in the development of cerebral malaria^46^. They expand early during plasmodial infection^47^ and human γδ^+^ T cells are the major producers of IFNγ upon *in vitro* re-stimulation with schizonts antigens^48^. Similarly we observed an early production of IFNγ by γδ^+^ T cells. Interestingly the early IFNγ production by γδ^+^ T cells was significantly increased in IL-22-deficient mice, which might lead to the lower parasitemia in IL-22-deficient mice. The lower parasitemia in the absence of IL-22 signalling was also found using infection with the PyNL strain, which indicate an effect of IL-22 independent of the plasmodial strain used. In the absence of the closely related cytokine IL-10 a similar result was already described^49^. The infection of IL-10-deficient mice with *P. chabaudi* lead to a lower parasitemia and an enhanced mortality^38^,^39^.

Also the transient neutralization of IL-22 by antibodies induced a similar phenotype as seen in *Il22*^−/−^ mice. This excludes that compensatory mechanisms in *Il22*^−/−^ mice are responsible for the observed phenotype. It is already known that IL-22 deficiency may alter the microbiome of mice and therefor may influence the course of malaria indirectly. However it is unlikely that the transient neutralization of IL-22 alters the microbiome till the occurrence of cerebral symptoms^25^,^26^.

It is known that IL-10-defiency leads to hepatic pathology during murine malaria^40^. Thus liver damage was analysed in *Il22*^−/−^ mice. Even though hepatocytes express high levels of the IL-22Rα1 chain^29^, we observed no different levels of liver transaminases in PbA-infected wt and *Il22*^−/−^ mice. Moreover IL-10 is known to have a regulatory effect on development of T_H_1 cell^50^,^51^. Thus we analysed if the closely related cytokine IL-22^5^ may have similar effects on the T cell response during murine malaria. One key player during the antimalarial immune response is IFNγ which is the hallmark cytokine of a T_H_1 dominated immune response. The high IFNγ production of different splenic lymphocyte subsets at an early time point of PbA infection and the enhanced susceptibility for cerebral malaria symptoms in the absence of IL-22 might be due to an enhanced T_H_1 response during PbA infection^52^. Even though the IFNγ production is comparable between wt and *Il22*^−/−^ mice at d6 p.i., at d3 p.i. T cells from *Il22*^−/−^ mice produce significantly more IFNγ. Interestingly a significantly reduced production of IL-17 is detectable regardless of the time point after infection. The decreased IL-17 production in *Il22*^−/−^ mice might be a consequence of the early increased IFNγ production by T cells in the absence of IL-22, since IFNγ is known to suppress T_H_17 differentiation^53^,^54^. Hence we assume that the T_H_1 differentiation is more favoured in IL-22-deficient mice. This increased IFNγ production was also observed in the supernatant of antigen-specifically stimulated splenocytes from PbA-infected *Il22*^−/−^ mice using PbA-specific peptides^55^,^56^. To further identify the reasons for an increased activation of T cells we studied the function of antigen-presenting cells. Interestingly, CD8^+^ T cells from PbA-infected *Il22*^−/−^ mice showed already a higher IFNγ production upon re-stimulation with wt BMDCs compared to CD8^+^ T cells of PbA-infected wt mice. This difference in IFNγ production was even more pronounced if the CD8^+^ T cells were re-stimulated with BMDCs of *Il22*^−/−^ mice. Moreover, the re-stimulation of CD8^+^ T cells by BMDCs generated from *Il22*^−/−^ mice induced a stronger IFNγ release regardless of the origin of the CD8^+^ T cells. This finding goes along with the observations that the transient neutralization of IL-22 in the beginning of plasmodial infection leads to the same malaria pathology, concluding that IL-22 has an influence on the activation of the adaptive immune system. The increased ability to stimulate T cells of *Il22*^−/−^ BMDC might be explained by their increased expression of the costimulatory molecule CD86 on dendritic cells in the spleens of PbA-infected *Il22*^−/−^ mice compared to wt mice. Thus IL-22 may dampen directly or indirectly the expression of costimulatory ligands of DCs during PbA infection. However, the addition of recombinant IL-22 during LPS stimulation of BMDCs was unable to inhibit the expression of CD86 and CD80, which might be due to an overly strong stimulatory effect of LPS. In addition in was shown recently that several subtypes of DCs produce IL-22BP constitutively^10^, this might interfere with the direct interaction between IL-22 and BMDCs during this experiment. An alternative explanation would be that IL-22 influences maturation of DCs indirectly by inducing factors on yet unknown cells that interfere with DCs. Interestingly, using the infection with *Listeria monocytogenes* no increase of the antigen-specific T cell response in *Il22*^−/−^ mice was observed^24^. However in this infection model it remains unclear if IL-22 is induced and the absence of any effect of IL-22-deficieny on adaptive immunity might be due the lack of IL-22-induced signalling during *Listeria monocytogenes* infection.

Considering the enhanced IFNγ release and the increased expression of costimulatory ligands by DCs, it was of interest if the proliferation of PbA-OVAtg specific CD8^+^ T cells *in vivo* is improved in the absence of IL-22. Even though the proliferation of CFSE^+^ CD8^+^ T cells was highly synchronous in wt and *Il22*^−/−^ mice at d5 p.i. the fraction of cells that start to proliferate was higher in IL-22-deficient mice leading to the assumption that the lack of IL-22 leads to an increased antigen specific activation of T cells and to a better survival or an altered migration of activated T cells.

Taken together the low parasitemia and the earlier onset of cerebral malaria in the absence of IL-22 might be due to the enhanced expression of costimulatory receptors by DCs followed by an increased activation of antigen specific CD8^+^ T cells and the earlier IFNγ production of different lymphocyte subsets.

Nevertheless it remains unclear how IL-22 modulates the immune response during PbA infection. In other diseases the expression of the IL-22Rα1 chain was already shown on hematopoietic cells^3^,^4^ although it was for long postulated that these cells do not express the IL-22Rα1 chain^2^,^29^. Therefore the idea that cells of the immune system are completely absent of an IL-22Rα1 chain expression has to be reconsidered. However, further investigations are needed to elucidate that IL-22 directly acts on hematopoietic cells expressing the IL-22Rα1 chain during malaria. It is also possible that IL-22 interacts with non-hematopoietic cells and indirectly modulates the immune system via yet unidentified factors.

## Materials and Methods

### Ethics

C57BL/6J (wt), Tg(TeraTerb)1100MjB (OT1), NMRI and *Il22*^−/−^ mice were bred in the animal facility of the Bernhard Nocht Institute for Tropical Medicine. *Il22*^−/−^ mice were described previously^24^ and were used when backcrossed 12 times on C57BL/6 background. For all *Il22*^−/−^ and wt comparisons co-housed mice were used. For experiments using only wt mice, animals were purchased from Charles River (Sulzfeld, Germany). All animal experiments were performed in agreement with the German animal protection law under the supervision of a veterinarian. The responsible federal health Authorities of the State of Hamburg (Behörde für Gesundheit und Verbraucherschutz) has reviewed and approved the experimental protocol under the permission number 32/15. Mice were sacrificed by cardiocentesis under deep CO_2_ narcosis. All animals were used at the age of 7 to 10 weeks and were kept in individually ventilated cages under specific pathogen free conditions.

### Human samples

Plasma from 30 patients with acute *P. falciparum* malaria was used. These were obtained from travellers treated at the University Hospital Eppendorf in Hamburg. Samples were obtained after the first and fourth day of the treatment and display a parasitemia ranging from 0.01 to 5%. Diagnosis was performed in the diagnostic department of the Bernhard Nocht Institute for Tropical Medicine. 9 control samples were obtained from healthy donors. Ethical approval was obtained from the *Ethikkommission* Hamburg and all experiments were carried out in accordance with the relevant guidelines. Written informed consent was obtained from all enrolled participants prior to inclusion in the study.

### *Plasmodium* parasites and infections

PbA and PyNL strains were stored in liquid nitrogen in a solution containing 0.9% NaCl, 4.6% sorbitol and 35% glycerol. Pb-GFP and Pb-OVAtg were a kind gift of W.R. Heath^57^. To start an experiment, mice were injected with the respective *Plasmodium* strain intraperitoneal (i.p.) and the blood was collected after 5 to 6 days post infection (p.i.). Infected red blood cells (iRBCs) were subsequently used to infect the experimental mice with either 1 × 10^5^ iRBC PbA or 2 × 10^4^ iRBC PyNL i.p. To maintain the life cycle of plasmodia NMRI mice with a high parasitemia were anesthetized and used for a blood meal of *Anopheles stephensi* (*A. stephensi*) mosquito at the mosquito colony of the Bernhard Nocht Institute for Tropical Medicine. After 18 to 25 days the sporozoites were isolated by manual dissection of the salivary glands of mosquitos. For further experiments 1000 sporozoites were injected i.v. into each mouse. PbA-infected C57BL/6 mice develop cerebral malaria symptoms, which include the occurrence of neurological symptoms such as ataxia, convulsion and coma. These symptoms usually appear between day 6 and 8 p.i. and ultimately lead to dead. Mice developing cerebral symptoms were scored daily as described previously (score 0: no symptoms; score 1: deceleration and weight loss <10%; score 2: ruffled fur; score 3: weight loss <20%, strong deceleration, paralysis of one leg; score 4: coma; score 5: dead)^58^. To avoid unnecessary suffering, mice were euthanized when reaching score 3 and were registered as dead in Kaplan-Meier survival curve statistics, since mice developing score 3 never cleared symptoms in our model and would progress to score 5 within several hours. All experiments were in accordance with the local Animal Ethics Committee Regulations.

### Analysis of parasitemia

A small blood drop was collected from the tail vein to determine blood-stage parasitemia in a thin blood smear using WRIGHT stain. The sporozoite liver burden was analysed after 24 h. The livers of sporozoite-infected mice were perfused with PBS and subsequently frozen in liquid nitrogen. The RNA of whole liver was purified using TRIzol reagent (Life Technologies) and reversed transcribed into cDNA using the Maxima First Strand cDNA Kit (Thermo Scientific). The sporozoite liver burden was analysed via an 18S qPCR specific for plasmodial ribosomal RNA (18S_primer fwd: 5′-ggtgtattcdctttatttaatgctt-3, 18S_primer rev: 5′-cacgcgtgcagcctagtat-3′; GAPDH_primer fwd: 5′-ggggcgtgcagcctagtat-3′, GAPDH_primer rev: 5′-ccttccacaatgccaaagtt-3′)^59^. The quantification of the sporozoite liver load was determined using a SYBR Green qPCR (Thermo Scientific Maxima SYBR Green qPCR Master Mix) with following conditions: T_initial DNA denaturation_ = 95 °C, 900 sec; (T_DNA denaturation_ = 95 °C, 15 sec; T_primer annealing_ = 50 °C, 20 sec; T_elongation_ = 68 °C, 20 sec) × 35; T_final elongation_ = 72 °C, 30 sec; melting curve.

### Analysis of transaminases

PbA-infected mice were sacrificed at d6 p.i., the blood was collected in EDTA coated tubes and centrifuged at 5000 rpm, 15 min at RT. The plasma was stored at −20 °C. ALT and AST levels out from the plasma were analysed in 1 to 3 dilution in PBS with the Reflotron test system (Roche).

### Antibodies and flow cytometry

IL-22 neutralization *in vivo* was performed using 20 μg αIL-22 (R&D, polyclonal goat IgG) i.p. per mouse at d0 p.i. Control mice were injected with the corresponding goat IgG (R&D) or with PBS. Mice were infected with 1 × 10^5^ PbA iRBC i.p. and sacrificed at d3 p.i. For flow cytometry analysis, single-cell suspensions of splenic cells were obtained by mashing spleens through a 70 μm sieve into complete DMEM medium including 10% foetal calf serum, 1% L-glutamine and 0.5% penicillin/streptavidin. Erythrocyte lysis was performed subsequently for 5 min at RT. 2 × 10^6 ^cells were stimulated in the presence of 50 ng/mL PMA and 500 ng/mL Ionomycin for four hours including the last three hours with 2 μM Monensin for the detection of IL-17 and IFNγ and for six hours including the last five hours with 2 μM Monensin for the detection of IL-22. Intracellular stainings were performed using the Foxp3 transcription factor staining buffer (eBioscience). The following antibodies were used for flow cytometry analysis: αCD3 PerCP Cy5.5 (clone 145-2C11, Biolegend), αCD8 eFluor 450 (clone 53-6.7, eBioscience), αCD11b V450 (clone M1/70, BD Horizon), αCD11c APC (clone N418, Biolegend), αCD11c PE-Cy7 (clone N418, Biolegend), αCD80 FITC (clone 16-10A1, BD Pharmingen), αCD86 APC (clone GL-1, Biolegend), α γδT PE-Cy7 (clone GL3 Biolegend), αIFNγ PE (clone XMG 1.2, eBioscience), αIL-17 APC (clone 17B7, eBioscience), αIL-22 PE (clone 1H8PWSR, eBioscience), αNK1.1 Alexa Fluor 488 (clone PK 136, BD Pharmingen). Samples were measured on a BD LSRII or a BD FACSAria III. FACS data were analysed using the software FlowJo.

### CFSE proliferation assay

Single cell suspension of OT1 splenocytes were incubated with 1 μM CFSE for 2 min 40 sec and subsequently washed twice with PBS. 2 × 10^7^ OT1 splenocytes were transferred i.v. After 4 hours the mice were infected with 1 × 10^5^ PbA iRBC i.p. and sacrificed at d5 p.i. Splenocytes were stained with αCD8 eFluor 450 (clone 53-6.7, eBioscience) and analysed at the LSR II regarding their CFSE dilution.

### Cytokine analysis

PBS or PbA-infected mice were sacrificed at d6 p.i. and blood was collected via heart puncture in EDTA coated tubes. The blood plasma was obtained after centrifugation at 5000 rpm 15 min at RT and stored at −20 °C. The blood of *P. falciparum*-infected human individuals was centrifuged at 5000 rpm for 15 min at RT and stored at −20 °C. The human or murine IL-22 ELISA (eBioscience) was performed according to instructions of the manufacturer. Murine and human plasma samples were used in a 1 to 5 dilution and the OD was measured at 450 nm. Splenocytes of infected mice were stimulated with 1 μg/mL of a mixture of PbA peptides (Pb1: SQLLNAKYL; Pb2: IITDFENL; F4: EIYIFTNI)^55^,^56^ (Jerini Biotools) for 24 h at 37 °C and 9% CO_2_ in complete DMEM medium. The supernatant was stored at −20 °C. The IFNγ concentration in collected supernatants was analysed with the DuoSet ELISA development kit (R&D Systems).

### Preparation of bone marrow derived dendritic cells (BMDCs)

Cells were collected out of the femur and tibia. Subsequently, these cells were treated with erythrocyte lysis buffer, the cell number determined and 3 × 10^6 ^cells were seeded per 10 cm dish. Bone marrow derived dendritic cells were generated in complete DMEM medium in the presence of granulocyte macrophage colony-stimulating factor (GM-CSF) for 7 days. GM-CSF containing complete DMEM medium was added to the culture each third day additionally. At d7 BMDCs were used for further experiments.

### BMDCs stimulation

1 × 10^5^ BMDCs were seeded in uncoated polystyrol well of a 96 well plate and stimulated with 0.1 μg/mL LPS (Sigma-Aldrich) with or without 0.05 μg/mL rIL-22 (PeproTech) for 24 h in complete DMEM medium at 37 °C and 9% CO_2_. BMDCs were subsequently collected and prepared for FACS staining.

### *In vitro* CD8^+^ T cell stimulation

2000 BMDCs were seeded per well in a 96 round bottom-plate and pulsed with 1 μg/mL PbA Peptide for 4 h in complete DMEM medium at 37 °C and 9% CO_2_. After one washing step with PBS, 5 × 10^4^ splenic CD8^+^ T cells, which were enriched through magnetic cell separation (MACS, Miltenyi) to a purity of >95%, were added to each well. The culture was incubated for 24 h at 37 °C at 9% CO_2_ in complete DMEM medium. The supernatant was stored at −20 °C for further analysis.

### Isolation of primary Hepatocytes

Primary Hepatocytes (pHepa) were isolated as described in Burghardt *et al.*^60^. Subsequently isolated cells were stored in TRIzol reagent (Life Technologies) at −70 °C.

### Statistical analysis

The GraphPad Prism 5 software was used to perform statistical analysis. Statistical differences in survival were analysed using Kaplan-Meier survival curve statistics. Additional statistical differences were identified using an unpaired t-test. For parasitemia testing the Mann-Whitney test was performed, assuming no Gaussian distribution for tested experiments. A regular two way ANOVA with a Bonferroni post-test was performed for pooled data. *p*-Values were indicated as *p < 0.05; **p < 0.01; ***p < 0.001. All experiments were performed at least twice and indicated error bars are based on SEM values.

## Additional Information

**How to cite this article**: Sellau, J. *et al.* IL-22 dampens the T cell response in experimental malaria. *Sci. Rep.* **6**, 28058; doi: 10.1038/srep28058 (2016).
